# Effect of ITPA Polymorphism on Adverse Drug Reactions of 6-Mercaptopurine in Pediatric Patients with Acute Lymphoblastic Leukemia: A Systematic Review and Meta-Analysis

**DOI:** 10.3390/ph15040416

**Published:** 2022-03-29

**Authors:** Yeonhong Lee, Eun Jeong Jang, Ha-Young Yoon, Jeong Yee, Hye-Sun Gwak

**Affiliations:** 1 College of Pharmacy and Graduate School of Pharmaceutical Sciences, Ewha Womans University, Seoul 03760, Korea; yhlee@ncc.re.kr (Y.L.); heimdall01@hanmail.net (E.J.J.); hayoungdymphnayoon@gmail.com (H.-Y.Y.); jjjhello1@naver.com (J.Y.); 2 Department of Pharmacy, National Cancer Center, Goyang-si 10408, Korea

**Keywords:** 6-mercaptopurine, inosine triphosphate pyrophosphatase, ITPA 94C>A, polymorphism, adverse drug reactions

## Abstract

6-Mercaptopurine (6-MP) is a cornerstone of the maintenance regimen for pediatric acute lymphoblastic leukemia (ALL). Inosine triphosphate pyrophosphatase (ITPA) is considered a candidate pharmacogenetic marker that may affect metabolism and 6-MP-induced toxicities; however, the findings are inconsistent. Therefore, we attempted to evaluate the effect of ITPA 94C>A polymorphism on 6-MP-induced hematological toxicity and hepatotoxicity through a systematic review and meta-analysis. A literature search for qualifying studies was conducted using the PubMed, Web of Science, and Embase databases until October 2021. Overall, 10 eligible studies with 1072 pediatric ALL patients were included in this meta-analysis. The results indicated that ITPA 94C>A was significantly associated with 6-MP-induced neutropenia (OR 2.38, 95% CI: 1.56–3.62; *p* = 0.005) and hepatotoxicity (OR 1.98, 95% CI: 1.32–2.95; *p* = 0.0009); however, no significant association was found between the ITPA 94C>A variant and 6-MP-induced leukopenia (OR 1.75, 95% CI: 0.74–4.12; *p* = 0.20). This meta-analysis demonstrated that ITPA 94C>A polymorphism could affect 6-MP-induced toxicities. Our findings suggested that ITPA genotyping might help predict 6-MP-induced myelosuppression and hepatotoxicity.

## 1. Introduction

Acute lymphoblastic leukemia (ALL) is the most common pediatric malignancy, accounting for approximately 25% of all cancers among children and 75–80% of childhood leukemias [1,2]. The survival rate and cure rate have improved over the past few decades with the optimal use of antileukemic drugs [3,4].

A combination of daily 6-mercaptopurine (6-MP) and weekly methotrexate for two to three years is the standard maintenance therapy for childhood ALL [5,6]. The inclusion of 6-MP has greatly improved the survival rate in leukemia therapy [7]. However, 6-MP has a narrow therapeutic index, especially in pediatric ALL patients, and exhibits dose-limiting toxicity in hematopoietic tissues [8]. Moreover, 6-MP exhibits large inter-individual variations in genetic polymorphisms responsible for metabolism, and some patients require dose reduction or treatment interruption due to adverse effects, including severe myelosuppression and hepatotoxicity, which can lead to life-threatening situations [9]. Recently, it has been found that polymorphisms in thiopurine methyltransferase (TPMP) and nudix hydrolase 15 (NUDT15) enzymes are involved in thiopurine metabolism associated with 6-MP-induced marrow suppression [10,11,12,13].

Inosine triphosphate pyrophosphatase (ITPA), another enzyme involved in purine metabolism, catalyzes the pyrophosphohydrolysis of inosine triphosphate (ITP) to inosine monophosphate (IMP). ITPA 94C>A (rs1127354) is one of the most well-known polymorphisms associated with ITPA deficiency, which traps purines in the form of ITP, resulting in thiopurine toxicities, including myelosuppression and hepatotoxicity. As ITPA plays a role in protecting cells from the accumulation of toxic metabolites, such as ITP, it has been considered as a possible candidate gene that may affect metabolism and 6-MP-induced toxicities with inter-individual variability [14,15,16].

Although several studies reported a clinical association between ITPA polymorphism and toxicities related to 6-MP treatment, the results were inconsistent. Therefore, we conducted a comprehensive systematic review and meta-analysis to determine the association between 94C>A polymorphism and 6-MP-induced toxicities in pediatric ALL. 

## 2. Methods

### 2.1. Literature Search and Strategy

This meta-analysis was conducted according to the Preferred Reporting Items for Systematic Reviews and Meta-analyses (PRISMA) guidelines [17]. The registration number is INPLASY202220110. Two researchers independently searched the literature using three databases (PubMed, Web of Science, and Embase). The following keywords were included: (mercaptopurin* OR 6-mercaptopurin* OR 6-mp OR purinethiol OR purinethol OR thiopurin* OR thiohypoxanthin*) AND {(acute lymphoblastic leukemia) OR (acute lymphoblastic leukaemia) OR ALL OR (lymphoblastic leukemia) OR (lymphoblastic leukaemia) OR (lymphoblastic lymphoma) OR (acute lymphocytic leukemia) OR (acute lymphocytic leukaemia) OR (lymphoid leukemia) OR (lymphoid leukaemia)} AND {(inosine triphosphate pyrophosphatase) OR (inosine triphosphatase) OR (inosine triphosphate pyrophosphohydrolase) OR (ITP pyrophosphohydrolase) OR ITPase OR ITPA} AND (polymorph* OR variant* OR mutation* OR genotyp* OR phenotyp* OR haplotyp* OR allele* OR SNP* OR pharmacogen*). A literature search was conducted on 25 October 2021, and the references of searched articles were screened.

### 2.2. Inclusion and Exclusion Criteria

Studies were included if they met the following criteria: (1) patients diagnosed with pediatric ALL received 6-MP-based maintenance therapy; (2) evaluated the association between the toxicity of 6-MP and ITPA 94C>A polymorphism; (3) provided sufficient data to calculate the odds ratio (OR) and 95% confidence interval (CI). Studies were excluded due to the following reasons: (1) conference abstracts, summaries, and reviews; (2) unable to extract the data; (3) not written in English. Only the most recent and comprehensive data were included in this study if overlapping data were identified.

### 2.3. Data Extraction

Two researchers independently extracted all data, and inconsistencies were discussed and resolved by consensus. The following information was collected from each eligible study: first author’s name, publication year, country, number of patients receiving 6-MP (male %), mean age, 6-MP dose, definition of outcomes (leukopenia, neutropenia, and hepatotoxicity), and genotyping method. In addition, the number of patients with and without leukopenia, neutropenia, and hepatotoxicity were recorded for each study. Among the outcomes, data on febrile neutropenia were included in neutropenia. We requested some data from the corresponding authors when the data were not extractable from the published paper.

### 2.4. Quality Assessment

Two researchers conducted a quality assessment independently according to the Newcastle-Ottawa Scale (NOS) for cohort studies [18]. The scoring system of the NOS has three categories, including subject selection (0–4 points), comparability of study groups (0–2 points), and outcome (0–3 points), with a total score of 0–9 points. In this review, we rated 1 point for each item of comparability if age and other known risk factors (such as sex) were matched or adjusted for in the analysis.

### 2.5. Statistical Analysis

Meta-analysis was performed using Review Manager (RevMan) version 5.4 (The Cochrane Collaboration, Copenhagen, Denmark). The OR and 95% CI were used to determine the association between ITPA 94C>A polymorphism and risk of 6-MP-induced toxicities. A *p* value < 0.05 was considered statistically significant. Heterogeneity between studies was estimated with the chi-square test and I^2^ statistic. I² > 50% was regarded as statistically significant heterogeneity. The selection of the proper effects model was based on the analysis results. In the absence of any statistical evidence of heterogeneity, the fixed-effects model (Mantel-Haenszel method) was used; otherwise, the random-effects model (DerSimonian-Laird method) was used to calculate pooled estimates [19,20]. Sensitivity analysis by sequentially excluding each study and subgroup analysis by ethnicity were performed. Begg’s rank correlation test and Egger’s regression test for funnel plot to identify publication bias were performed using R Studio software version 3.6.0 (R Foundation for Statistical Computing, Vienna, Austria) [21,22]. 

## 3. Results

**Figure 1:**
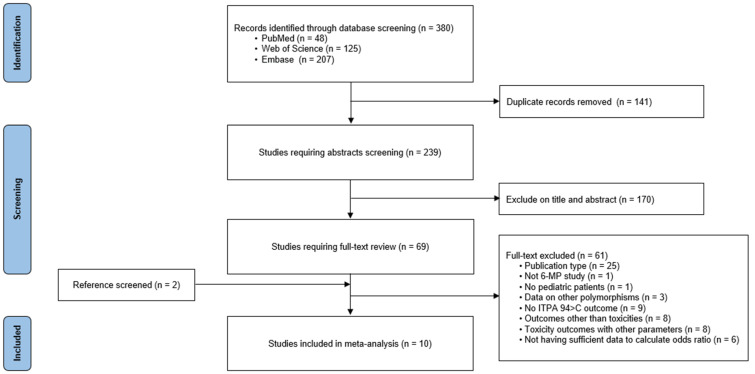
PRISMA flow diagram for the meta-analysis.

A detailed flow chart of the study selection process is shown in Figure 1. A total of 380 records were identified from three databases (PubMed = 48, Web of Science = 125, and Embase = 207). After the removal of 141 duplicates, 239 records remained. Among them, 170 studies were excluded based on the title and abstract, and 69 potentially relevant studies were eligible for full-text review. Of these studies, 61 studies were excluded for the following reasons: publication type (*n* = 25), not 6-MP study (*n* = 1), no pediatric patients (*n* = 1), data on other polymorphisms (*n* = 3), no ITPA 94C>A outcome (*n* = 9), outcomes other than toxicity (*n* = 8), toxicity outcomes with other parameters (*n* = 8), and not having sufficient data to calculate the OR (*n* = 6). An additional two studies were added through manual search. One study [23] involved two ethnicities (Kurds and Lebanese); data were extracted separately for each ethnicity. Ultimately, 10 studies including 11 datasets were included in this meta-analysis [23,24,25,26,27,28,29,30,31,32]. 

Table 1 summarizes the characteristics of the included studies. The studies were published between 2009 and 2021. Of 10 studies, a total of 6, 2, 1, and 1 studies were conducted in Asia [25,26,27,29,31,32], the Middle East [23,24], the USA [30], and Europe [28], respectively. The NOS score ranged from 6 to 8. 

**Figure 2:**
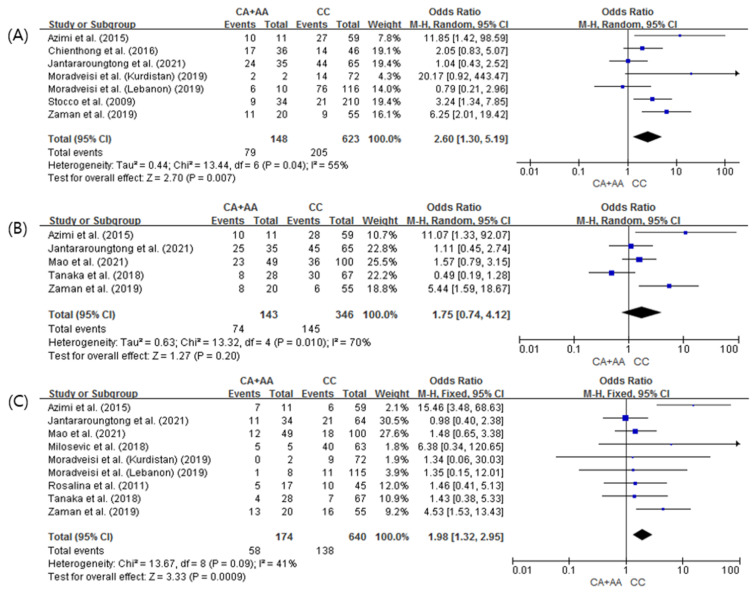
Forest plot of the association between ITPA 94C>A polymorphism and 6-MP-induced toxicities: (**A**) Neutropenia, (**B**) Leukopenia, and (**C**) Hepatotoxicity.

The meta-analysis results comparing the toxicities of 6-MP between the ITPA 94C>A variant (CA or AA) and wild-type homozygote (CC) groups are shown in Figure 2. A total of seven studies comprising a total of 771 patients with pediatric ALL were included for the analysis of neutropenia; in comparison with the wild-type homozygote group, the ITPA 94C>A variant group was significantly associated with an increased risk of neutropenia (OR 2.38, 95% CI: 1.56–3.62; *p* = 0.005). As there was heterogeneity among these studies (*I*^2^ = 55%, *p* = 0.04), a random-effects model was used (Figure 2A). For leukopenia, there was no significant difference between patients with the ITPA 94C>A variant allele and wild-type homozygous patients (OR 1.75, 95% CI: 0.74–4.12; *p* = 0.20) using a random-effects model (*I*^2^ = 70%, *p* = 0.01) (Figure 2B). For hepatotoxicity analysis, 9 studies with 814 patients were evaluated. Patients with the ITPA 94C>A variant allele had a significantly increased risk of hepatotoxicity compared with wild-type homozygous patients (OR 1.98, 95% CI: 1.32–2.95; *p* = 0.0009) using a fixed-effects model (*I*^2^ = 41%, *p* = 0.09).

Sensitivity analysis was performed to assess the stability of the results by sequential omission of each study (Table 2). According to the ORs, the results were similar for neutropenia (OR range: 2.16–3.11, *I*^2^ range: 46–63%). However, sensitivity analysis of leukopenia indicated that the ITPA 94C>A variant had significantly increased toxicity risk (OR 2.38, 95% CI: 1.02–5.52) with the omission of the Tanaka et al. study [25]. In addition, hepatotoxicity results showed an OR range of 1.37–2.41 with an *I*^2^ range of 0–49%. When the Azimi et al. study was excluded, heterogeneity was greatly reduced (*I*^2^ = 0%, *p* = 0.50) [21]. 

Subgroup analysis by ethnicity was also performed (Figure S1). There were no significant ethnic differences in the associations between ITPA 94C>A and 6-MP-induced toxicities (all *p* > 0.05). As the number of studies included in each analysis was limited, some results of subgroup analysis did not achieve statistical significance. For hepatotoxicity, 94C>A variant significantly increased the risk in Asians (OR: 1.6; 95% CI: 1.0–2.5) and Middle Eastern (OR: 5.1; 95% CI: 1.9–13.5).

**Figure 3:**
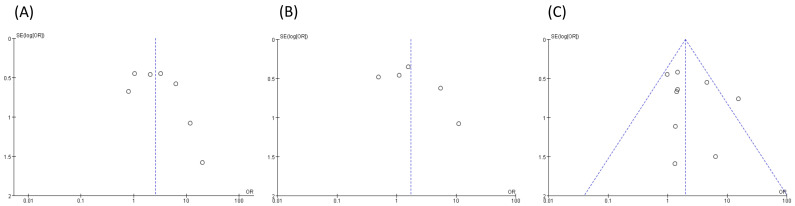
Funnel plot for publication bias of the included studies: (**A**) Neutropenia, (**B**) Leukopenia, and (**C**) Hepatotoxicity.

The funnel plots for outcomes are shown in Figure 3. The results of Begg’s test and Egger’s test indicated that there was no significant publication bias in studies of neutropenia (*p* = 0.2931 and *p* = 0.2415, respectively), leukopenia (*p* = 0.6242 and *p* = 0.3139, respectively), and hepatotoxicity (*p* = 0.1444 and *p* = 0.4146, respectively).

## 4. Discussion

This meta-analysis evaluated the association between ITPA gene polymorphism (94C>A) and 6-MP-induced toxicities in pediatric patients with ALL. Our results indicated that the 94C>A variant was significantly associated with an increased risk of neutropenia and hepatotoxicity. Sensitivity analysis demonstrated consistent results. 

Maintenance therapy is required to prevent relapse for patients with ALL, and prolonged exposure to 6-MP is an important part of the maintenance regimen [33]. 6-MP requires a multi-enzymatic process initiated by hypoxanthine-guanine phosphoribosyltransferase, which is converted to 6-thioinosine monophosphate, leading to the formation of the pharmacologically active metabolites, such as 6-thioguanine nucleotide (6-TGN). When 6-TGN is incorporated into DNA and RNA, it inhibits DNA synthesis, resulting in cytotoxicity [34,35,36]. 

ITPA catalyzes the hydrolysis of ITP to IMP. IMP is a key metabolite in purine metabolism, which is converted to adenosine triphosphate (ATP)/guanosine triphosphate (GTP) via adenosine monophosphate (AMP)/guanosine monophosphate (GMP) [16]. ITPA is a protective enzyme that prevents the accumulation of toxic metabolites, such as 6-thioinosine triphosphate, during 6-MP metabolism [37]. Among the five identified single nucleotide polymorphisms of ITPA, the ITPA 94C>A variant is associated with ITPase deficiency [16]. In vitro and in vivo studies indicated the ITPA 94C>A variant has around 50% of the enzymatic activity of the wild-type [38], and clinical data showed a complete deficiency and decreased enzymatic activity to 25% for variant-type homozygotes and heterozygotes, respectively [16]. Hence, patients with a nonfunctional variant allele of ITPA have lower ITPA enzymatic activity, leading to abnormal accumulation of potentially toxic metabolites in erythrocytes, which could be associated with 6-MP-induced toxicities [15,39].

Several studies have reported that the ITPA 94C>A variant could increase the risk of thiopurine-related hematological toxicity [40,41,42,43], hepatotoxicity [44,45], flu-like symptoms [46], pancreatitis, and rash [46] in patients with pediatric ALL and inflammatory bowel disease (IBD), which is consistent with our results. In addition, it has been reported that the decreased activity of the ITPA enzyme is associated with a high level of methylated thiopurine nucleotides [14,45,47], known to have cytotoxic properties that may lead to hepatotoxicity [44,45]. 

Nevertheless, it was reported that the ITPA 94C>A variant has a protective mechanism against ribavirin (RBV) toxicity. RBV is a purine nucleoside analog that mimics inosine, guanosine, or adenosine [48] and exhibits antiviral activity after intracellular phosphorylation [49]. RBV-induced anemia is presumed to result from the depletion of ATP caused by GTP consumption when RBV is phosphorylated to ribavirin triphosphate, the active form of RBV [50]. As ITPA deficiency causes the accumulation of ITP, which can be used to synthesize ATP, the ITPA 94C>A variant reduces the incidence of RBV-induced anemia [51]. 

The allele frequencies of the ITPA 94C>A variant indicated inter-ethnic variability (1–2% in the Hispanic population, 5–7% in the Caucasian and African population, and 19% in the Asian population). In comparison with TPMP, the ITPA 94C>A allele shows an almost complete reversal in allele frequencies in each population [52,53]. Therefore, ITPA variants may be essential for predicting 6-MP-induced toxicities in Asians with a low frequency of TPMP variants. 

Previously, two meta-analyses published in 2007 and 2022 investigated the correlation between ITPA and the adverse effects of azathioprine (AZA)/6-MP. van Dieren et al. [54] did not demonstrate an association between ITPA and the development of thiopurine toxicities in patients with adult IBD, whereas Barba et al. [55] indicated that ITPA 94C>A was associated with adverse effects in the general adult population and neutropenia in pediatric patients with ALL.

Barba et al. [55] conducted a meta-analysis of the overall toxicity of azathioprine (AZA)/6-MP in all age groups. Subgroup analysis using pediatric ALL patients was performed in the meta-analysis, and the results for neutropenia were consistent with the findings of our study. However, among the 10 studies included in the subgroup analysis of patients with pediatric ALL for overall toxicity in the meta-analysis, only 2 studies overlapped with our study. In addition, there were only two studies each for the analysis of neutropenia, leukopenia, and hepatotoxicity in the previous meta-analysis. In contrast, there were at least five studies in our meta-analysis. 

Several limitations should be considered in this study. First, differences in study characteristics, including ethnicity and definition of toxicities, may lead to heterogeneity. Second, some confounding factors that could affect the risk of 6-MP-induced toxicities, such as maintenance regimens (e.g., 6-MP dosage and concomitant drugs), types of remission therapy, baseline lab values (e.g., white blood cell count, aspartate aminotransferase, alanine aminotransferase), and comorbidities, could not be adjusted. 

## 5. Conclusions

Despite the inconsistencies in individual results, we found that ITPA 94C>A polymorphism may be associated with an increased risk of 6-MP-induced neutropenia and hepatotoxicity. Therefore, our findings suggest that ITPA genotyping may help predict 6-MP-induced toxicities in patients with pediatric ALL. Nevertheless, the results should be confirmed with a larger population. 

## Figures and Tables

**Table 1:** Characteristics of studies included in the meta-analysis.

Study	Country	Sample Size (Male %)	Age (Years) (Mean ± SD)	6-MP Dose	Dose Adjustment	Concomitant Drugs	Outcome	Genotyping Method	NOS Score
Azimi et al. (2015) [24]	Iran	70 (48.6)	1–9 ^c^	50 mg/m^2^	To maintain a WBC count of 2000–3000/μL	MTX	Leukopenia Neutropenia Hepatotoxicity	Sanger method	7
Chiengthong et al. (2016) [25]	Thailand	82 (40.2)	5.4 (1–15) ^a^	50 mg/m^2^	To maintain ANC 500 -1500/μL	VCR, PD, MTX, IT MTX	ANC < 500/μL	Pyrosequencing	6
Jantararoungtong et al. (2021) [26]	Thailand	115 (54.8)	6.11 ± 3.86	75 mg/m^2^	To maintain WBC ≥ 1500/μL, ANC ≥ 500/μL ± infection records	Low risk: MTX 40 mg/m^2^ PO weekly VCR 2 mg/m^2^ IV monthly PD 40 mg/m^2^ PO 5 days/month Standard/high risk: MTX 40 mg/m^2^ PO weekly VCR 2 mg/m^2^ IV monthly PD 60 mg/m^2^ PO 5 days/month CP 300 mg/m^2^ IV monthly Ara-C 300 mg/m^2^ IV monthly	Leukopenia: WBC < 2000/μL Neutropenia: ANC < 1000/μL Hepatotoxicity: ALT > X 3 ULN	TaqMan assays	6
Mao et al. (2021) [27]	China	149 (57.0)	5.92 (0.63–13.75) ^a^	50 mg/m^2^	To maintain a WBC count of 2000–3000/μL	MTX 20 mg/m^2^ PO weekly VCR 1.5 mg/m^2^ IV monthly DEX 6 mg/m^2^ PO 5 days/month	Leukopenia: WBC < 2000/μL Hepatotoxicity: ALT > X 5 ULN	Fluorescence in situ hybridization	6
Milosevic et al. (2018) [28]	Serbia	60 (55.9)	5.2 (0.9-17.6) ^a^	50 mg/m^2^	To maintain a WBC count of 2000–3000/μL	MTX 20 mg/m^2^ PO weekly	Hepatotoxicity: Elevated levels of transaminases	PCR-RELP method	6
Moradveisi et al. (Kurdistan) (2019) [23]	Kurdistan	74 (58.1)	6.25 ± 3.07	75 mg/m^2^	To maintain a WBC count of 2000–3000/μL, ANC > 500/μL	MTX 20 mg/m^2^ PO weekly	Febrile neutropenia: ANC < 1000/mm^3^ with a single temperature of >38.3 °C (101 °F) or a sustained temperature of ≥38 °C (100.4 °F) for more than one hour Hepatotoxicity: ALT ≥ X 3 ULN	PCR-RELP method	6
Moradveisi et al. (Lebanon) (2019) [23]	Lebanon	136 (56.6)	6.63 ± 4.93	75 mg/m^2^	To maintain a WBC count of 1500–3000/μL, ANC > 300/μL, PLT > 50,000	MTX 40 mg/m^2^ PO weekly	Febrile neutropenia: ANC < 1000/mm^3^ with a single temperature of >38.3 °C (101 °F) or a sustained temperature of ≥38 °C (100.4 °F) for more than one hour Direct bilirubin ≥ 1.5	TaqMan allele	6
Rosalina et al. (2012) [29]	Malaysia	63 (52.3)	10.13 (1–20) ^b^	N/A	N/A	N/A	Liver toxicity	Allele-specific PCR	6
Stocco et al. (2009) [30]	USA	244 (58.6)	5.9 (0.08–18.8) ^a^	75 mg/m^2^	When patients developed toxicity attributable to 6-MP	Low risk: MTX 40 mg/m^2^ IV weekly DEX 8 mg/m^2^ PO 7 days/month VCR 1.5 mg/m^2^ IV monthly Higher risk: received drugs pairs rotating weekly ^d^	Grade 3/4 febrile neutropenia Grade 3: ANC < 1000/μL with a single temperature of >38.3 °C (101 °F) or a sustained temperature of ≥ 38 °C (100.4 °F) for more than one hour Grade 4: Life-threatening consequences; urgent intervention indicated	TaqMan assay	8
Tanaka et al. (2018) [31]	Japan	95 (49.5)	4.9 (1–17) ^a^	40 mg/m^2^	To maintain a WBC count of 2000–3500/μL	MTX 25 mg/m^2^ PO weekly	Leukopenia: WBC < 2000/μL or neutrophil count < 1000/μL Hepatotoxicity: ALT > 700 IU/L	TaqMan assays	6
Zaman et al. (2019) [32]	Bangladesh	75 (NA)	5 ± 2.5	75 mg/m^2^	When patients developed toxicity attributable to 6-MP	NA	Leukopenia: WBC < 3000/μL Neutropenia: ANC < 1000/μL Raised serum ALT: ALT > 36 U/L	TaqMan assays	8

Ara-C: cytarabine; ALT: alanine aminotransferase; ANC: absolute neutrophil count; CP: cyclophosphamide; DEX: dexamethasone; IT: intrathecal; MTX: methotrexate; NA: not applicable; NOS: Newcastle-Ottawa scale; PCR: polymerase chain reaction; PD: prednisolone; RELF: restriction fragment length polymorphism; SD: standard deviation; ULN: upper limits of normal; VCR: vincristine; VP-16: etoposide; WBC: white blood cell ^a^ median (range), ^b^ mean (range), ^c^ range, ^d^ Week 1: VP-16 300 mg/m^2^ IV + CP 300 mg/m^2^, Week 2: MTX 40 mg/m^2^ IV + 6-MP 75 mg/m^2^ PO daily, Week 3: MTX 40 mg/m^2^ IV + Ara-C 300 mg/m^2^, Week 4: VCR 1.5 mg/m^2^ + Dex 8 mg/m^2^ daily, Week 5: VP-16 300 mg/m^2^ IV + CP 300 mg/m^2^, Week 6: MTX 2000 mg/m^2^ + 6-MP 75 mg/m^2^ PO daily, Week 7: VP-16 300 mg/m^2^ IV + CP 300 mg/m^2^, Week 8: VCR 1.5 mg/m^2^ +Dex 8 mg/m^2^/ daily.

**Table 2:** Sensitivity analysis of the association between ITPA 94C>A status and 6-MP induced toxicities by sequentially excluding each study (ITPA wild type vs ITPA variant).

Study Excluded	Heterogeneity *I*^2^ (%)	Statistical Model	Odds Ratio (95% CI)
Neutropenia			
None	55	Random	2.60 (1.30–5.19)
Azimi et al. (2015)	55	Random	2.27 (1.14–4.54)
Chiengthong et al. (2016)	63	Random	2.87 (1.20–6.88)
Jantararoungtong et al. (2021)	46	Fixed	3.07 (1.90–4.96)
Moradveisi et al. (2019) (Kurdistan)	57	Random	2.36 (1.19–4.69)
Moradveisi et al. (2019) (Lebanon)	53	Random	3.11 (1.51–6.38)
Stocco et al. (2009)	61	Random	2.57 (1.09–6.06)
Zaman et al. (2019)	50	Random	2.16 (1.05–4.43)
Leukopenia			
None	70	Random	1.75 (0.74–4.12)
Azimi et al. (2015)	69	Random	1.39 (0.61–3.16)
Jantararoungtong et al. (2021)	77	Random	2.11 (0.67–6.71)
Mao et al. (2021)	77	Random	1.97 (0.56–6.89)
Tanaka et al. (2018)	58	Random	2.38 (1.02–5.52)
Zaman et al. (2019)	64	Random	1.30 (0.56–3.03)
Hepatotoxicity			
None	41	Fixed	1.98 (1.32–2.95)
Azimi et al. (2015)	0	Fixed	1.68 (1.10–2.58)
Jantararoungtong et al. (2021)	34	Fixed	2.41 (1.53–3.80)
Mao et al. (2021)	46	Fixed	1.37 (1.37–3.44)
Milosevic et al. (2018)	46	Fixed	1.90 (1.26–2.85)
Moradveisi et al. (2019) (Kurdistan)	49	Fixed	1.99 (1.33–2.98)
Moradveisi et al. (2019) (Lebanon)	48	Fixed	2.00 (1.33–3.01)
Rosalina et al. (2012)	48	Fixed	2.05 (1.34–3.13)
Tanaka et al. (2018)	48	Fixed	2.04 (1.34–3.12)
Zaman et al. (2019)	36	Fixed	1.72 (1.11–2.66)

## Data Availability

Data sharing not applicable.

## Supplementary Materials

The following supporting information can be downloaded at https://www.mdpi.com/article/10.3390/ph15040416/s1. Figure S1: Forest plot of subgroup analysis by ethnicity on the association between ITPA 94C>A polymorphism and 6-MP-induced toxicities: (A) Neutropenia, (B) Leukopenia, and (C) Hepatotoxicity.
