# Supplementary material for: Effect of ITPA Polymorphism on Adverse Drug Reactions of 6-Mercaptopurine in Pediatric Patients with Acute Lymphoblastic Leukemia: A Systematic Review and Meta-Analysis
Source: Pharmaceuticals (Basel). 2022 Mar 29;15(4):416. doi: 10.3390/ph15040416 (PMC9027773; doi:10.3390/ph15040416)

Figure S1: Forest plot of subgroup analysis by ethnicity on the association between ITPA 94C>A polymorphism and 6-MP-induced toxicities. (A) Neutropenia, (B) Leukopenia, (C) Hepatotoxicity.

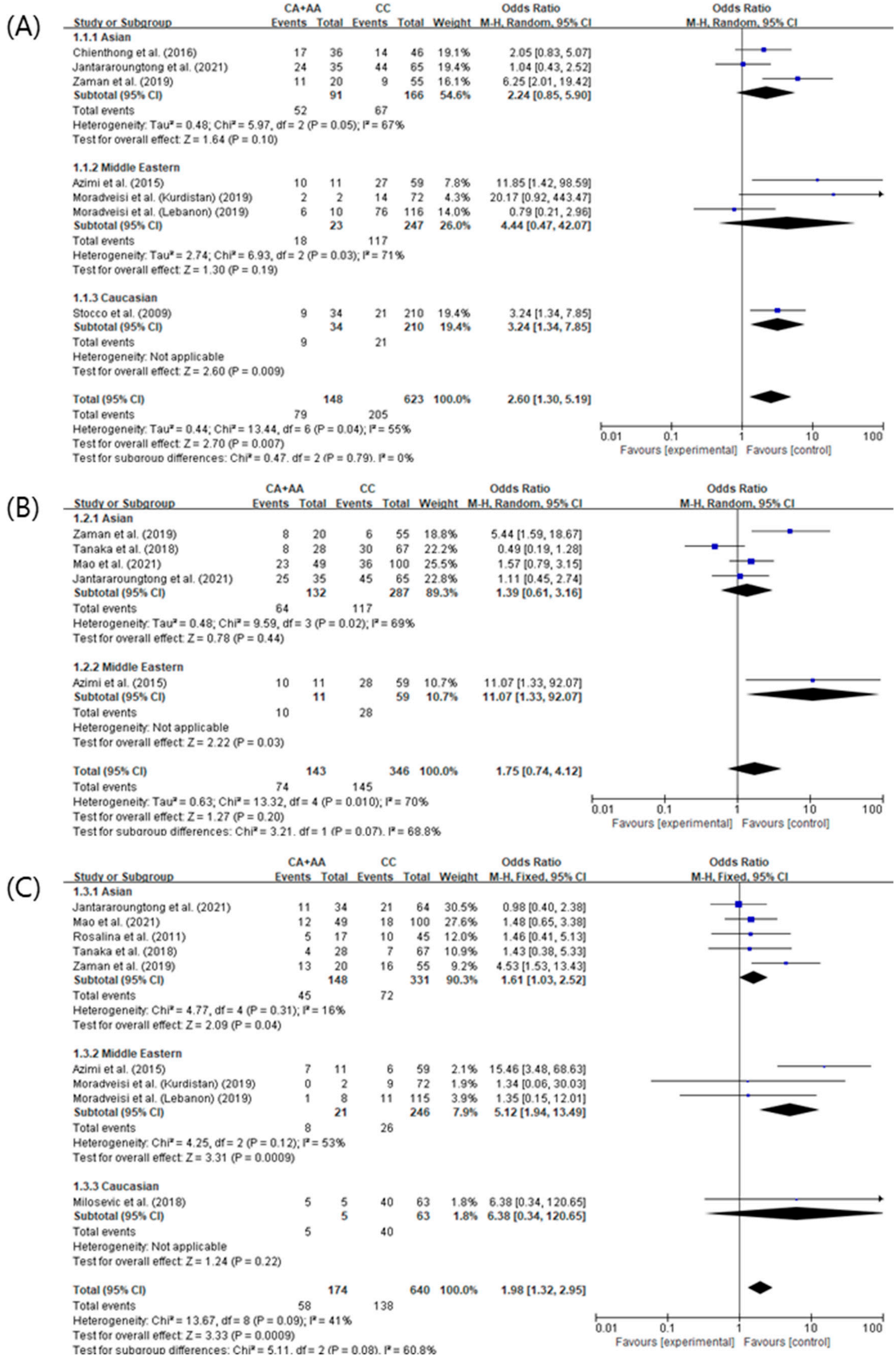

Supplement: Supplementary file 1 [file pharmaceuticals-15-00416-s001.zip › pharmaceuticals-1613775-supplementary.pdf]
